# Genetic diversity and phylogeny of South African *Meloidogyne* populations using genotyping by sequencing

**DOI:** 10.1038/s41598-018-31963-9

**Published:** 2018-09-14

**Authors:** Milad Rashidifard, Hendrika Fourie, Pierre-Yves Véronneau, Mariette Marais, Mieke Stefanie Daneel, Benjamin Mimee

**Affiliations:** 1 Unit for Environmental Sciences and Management, North-West University, Private Bag X6001, 2520 Potchefstroom, South Africa; 2 Agriculture and Agri-Food Canada, St-Jean-sur-Richelieu Research and Development Centre, 430 boul. Gouin, St-Jean-sur-Richelieu J3B 3E6 Québec, Canada; 3 Nematology Unit, Biosystematics, Agricultural Research Council - Plant Health and Protection (ARC-PHP), Private Bag X134, 0121 Queenswood, South Africa; 4 Agricultural Research Council - Tropical and Subtropical Crops (ARC – TSC), Private Bag X11208, 1200 Mbombela, South Africa

## Abstract

*Meloidogyne* species cause great crop losses worldwide. Although genetic host plant resistance is an effective control strategy to minimize damage caused by *Meloidogyne*, some resistant genes are ineffective against virulent species such as *Meloidogyne enterolobii*. Detailed knowledge about the genetic composition of *Meloidogyne* species is thus essential. This study focused on genotyping-by-sequencing (GBS) and Pool-Seq to elucidate the genetic relation between South African *M. enterolobii*, *M. incognita* and *M. javanica* populations. Hence, 653 common single nucleotide polymorphisms (SNPs) were identified and used to compare these species at genetic level. Allele frequencies of 34 SNPs consistently differed between the three *Meloidogyne* species studied. Principal component and phylogenetic analyses grouped the *M. enterolobii* populations in one clade, showing a distant relation to the *M. javanica* populations. These two species also shared genetic links with the *M. incognita* populations studied. GBS has been used successfully in this study to identify SNPs that discriminated among the three *Meloidogyne* species investigated. Alleles, only occurring in the genome of *M. enterolobii* and located in genes involved in virulence in other animal species (e.g. a serine/threonine phosphatase and zinc finger) have also been identified, accentuating the value of GBS in future studies of this nature.

## Introduction

Root-knot nematodes (*Meloidogyne*) are polyphagous, obligate pests that are distributed worldwide and parasitize almost all the higher plant species, resulting in great economic losses^1^. *Meloidogyne incognita* is generally considered as the most damaging root-knot nematode species worldwide^2^. Since this species can infect *Arabidopsis thaliana*, it is also a key model system to study metazoan adaptations to plant parasitism, hence its genome has already been elucidated^3^. However, *Meloidogyne enterolobii* listed as a threat species, can be confused with *M. incognita* and other thermophilic species due to it exhibiting similar morphological characteristics^4^. Of more significance is that *M. enterolobii* has the ability to overcome resistance genes that are effective against its thermophilic counterparts *Meloidogyne arenaria, M. incognita* and *Meloidogyne javanica*^5–8^. *Meloidogyne enterolobii* for example had been reported to reproduce optimally on tomato and pepper that exhibit the *Mi-*1*, N* and *Tabasco* resistance genes, respectively^8^, while *M. arenaria* failed to reproduce on such resistant plants^9^. This phenomenon has far reaching implications for the management of this species.

Management of root-knot nematodes has been done traditionally by means of chemical control. This approach generally keeps the nematode population under the economic threshold level since eradicating these pests is considered impossible^4,10^. However, the development of resistance against the different chemical compounds and the progressive withdrawal of synthetically-derived nematicides due to animal, human and environmental concerns^11,12^ are the main drives for the exploitation and use of environmentally-friendly strategies. Currently, genetic host plant resistance is a very effective and viable strategy to control root-knot nematodes in various agricultural cropping systems^13^. Nonetheless, the existence of virulent root-knot nematode populations reduces the efficacy of this strategy^14^. *Meloidogyne enterolobii* occurs in many countries and has initially been reported from the Mpumalanga Province in South Africa during the 1990s from guava (*Psidium guajava*) orchards^15^. Its established occurrence in South Africa fits the hypothesis of the late Dr Kent Kleynhans and Mr Piet Willers that the occurrence and host range is wider than the initial localities and hosts around Mbombela, Mpumalanga (personal communication, Dr Kent Keynhans, Agricultural Research Council-Plant Protection Research Institute, Pretoria, 1998). It has hence been reported from other crop production areas, infecting green pepper (*Capsicum annuum*); potato (*Solanum tuberosum*) and tomato (*Solanum lycopersicum*)^16,17^. This scenario justified a more detailed genetic study of South African *Meloidogyne* populations to determine if genomic differences linked with virulence exhibited by *M. enterolobii* could be found between *M. incognita* and *M. javanica*.

Several studies have been conducted to elucidate the genetic diversity of *Meloidogyne* populations by using different molecular techniques, e.g. random amplified polymorphic DNA (RAPD), restriction fragment length polymorphisms (RFLP), PCR based on sequences of rDNA, mtDNA, ITS and IGS, or satellite DNA probe markers^18–26^. However, most of these methods are expensive, time-consuming, require several PCR analyses and many nematode individuals^27^. The ultimate drawback of these techniques are that they are targeting only a small part of the genome of a nematode and are hence not optimal for pan-genomic comparison. It is therefore necessary to apply novel and rapid molecular genotyping tools to obtain more detailed information about the genetic diversity between *Meloidogyne* species.

Single nucleotide polymorphisms (SNPs) are popular and common molecular markers used to study the entire genome of nematodes^28,29^. Significant advances in sequencing technologies are providing lots of information at relatively low cost^30^. Genotyping by sequencing (GBS)^31^ is a simple protocol based on next generation-sequencing (NGS) of genomic fragments of organisms (e.g. nematodes) obtained by specific restriction enzymes followed by a bioinformatics pipeline^30^. This enzyme-based reduction of complexity, combined with the use of barcodes for multiplexing considerably reduces sequencing cost while providing genome-wide information^32^. This technique has proven to be useful and accurate to characterize nematode species even when no information about the genome was available^27^. Currently, good reference genomes are available for *M. hapla*^33^ and *M. incognita*^3,34^. However, no annotated reference genomes exist for *M. enterolobii* and *M. javanica*, although some assemblies from whole genome sequencing (WGS) data were published recently^34,35^. Genotyping by sequencing has, for example successfully been applied in combination with Pool-Seq by Mimee *et al*.^27^, to investigate the genetic diversity among populations of the golden cyst nematode *Globodera rostochiensis*. Pool-Seq is a method described by Futschik and Schlötterer^36^, which instead of sequencing isolated individuals directly uses a population (several individuals pooled together). When using a sufficiently big pool size, Pool-Seq even showed to be more appropriate for estimating allele frequencies and is more cost effective than sequencing the DNA of individuals^36^.

This study aimed to investigate the genetic diversity of three different *Meloidogyne* species *viz. M. enterolobii*, *M. incognita* and *M. javanica* using GBS in order to highlight relationships among these species and loci putatively involved in virulence.

## Results

### SNP calling

The sequencing of 11 *Meloidogyne* populations digested with *Pst*I/*Msp*I restriction enzymes generated 83 038 291 reads. After initial quality control, 77 095 925 good barcoded reads were kept for further analysis. The UNEAK pipeline identified 2,786 SNPs before filtering. The final dataset contained from 59 to 929 SNPs depending on filtering stringency (Table 1). In order to ensure a good accuracy, the dataset without missing data containing 277 SNPs (minimum call rate = 1.0 and, minimum coverage = 20) was kept for phylogenetic and Principal Component Analyses (PCA). The dataset, containing 653 SNPs, was explored to find interesting markers. The allele frequencies at these loci in the 11 *Meloidogyne* populations are presented in Supplementary Table 1. When the pipeline was run on the *M. enterolobii* populations only, 13,047 SNPs were identified. Of these, 2,092 were present in all populations and supported by a minimum coverage of 20 reads (Table 1).

**Table 1:** Influence of the minimum call rate (mnC) and the minimum coverage at each locus (minCov) on the number of SNPs identified by the UNEAK pipeline for *Meloidogyne enterolobii*, *M. incognita* and *M. javanica* populations from South Africa.

	mnC	minCov		
		5	20	50
11 populations	0.8	929	542	140
	1.0	653	277	59
*M. enterolobii *only	0.7	7,534	2,683	1,324
	1.0	5,572	2,092	1,032

A total of 1,016 variants were called after alignment of the raw reads to the *M. incognita* genome and 419 of them were in predicted genes. However, very few remained after filtering. For a minimum call rate of 1.0, only 95, 84 and 64 SNPs had a minimum coverage of 5, 20 and 50 reads, respectively, and 148, 122 and 93 SNPs when a minimum call rate of 0.8 was used with the same coverage.

### Population genetics

**Figure 1:**
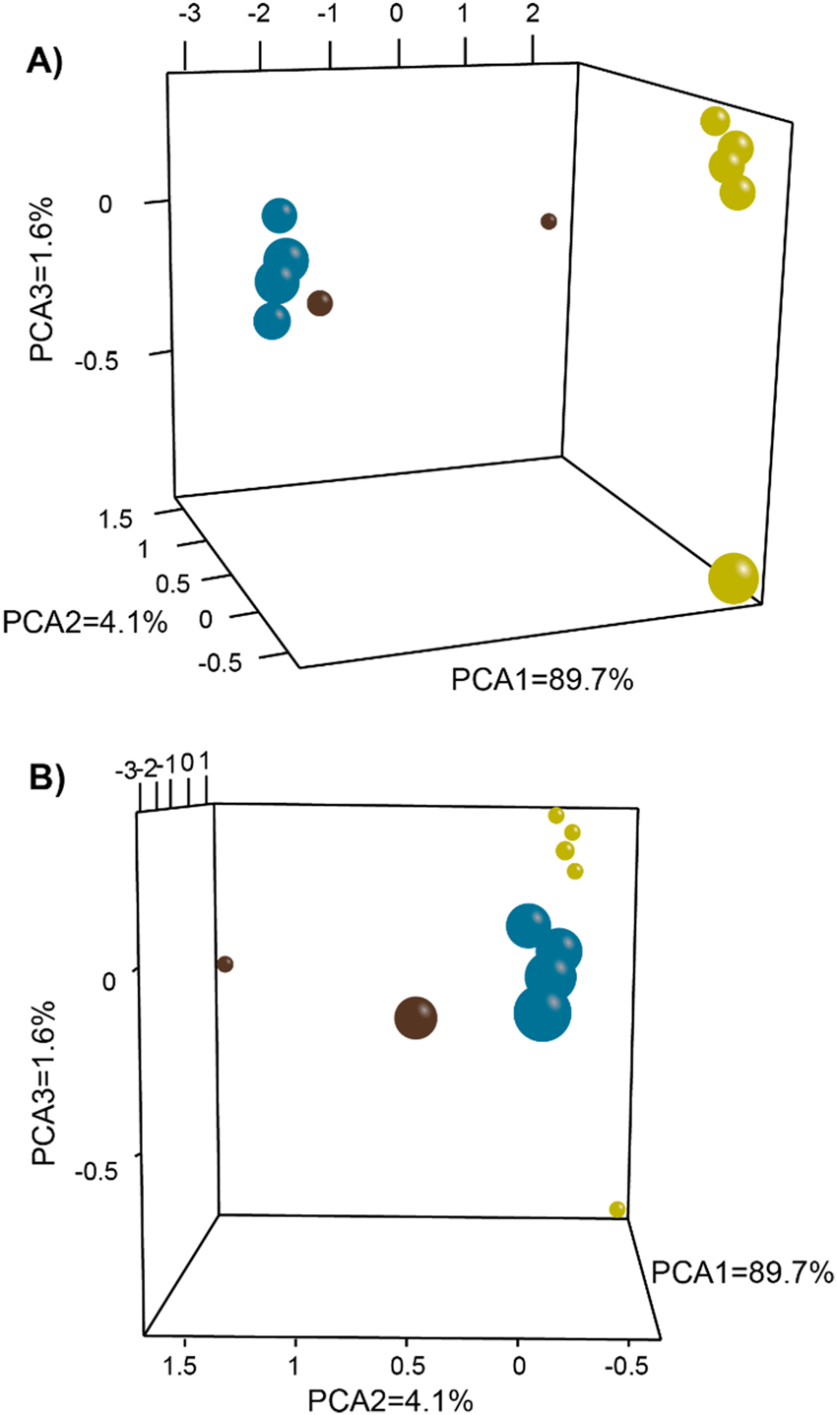
Principal Component Analysis (PCA) of 11 South African *Meloidogyne* populations based on allele frequencies at 277 loci and showing the genetic relations among two *M. incognita* (brown spheres), four *M. enterolobii* (blue spheres), and five *M. javanica* (beige spheres) populations. Panels show (**A**) First dimension on x axis and (**B**) second dimension on x axis.

PCA of allele frequencies of the 11 *Meloidogyne* populations using the 277 SNPs dataset separated the populations in two main clusters (Fig. 1). All the *M. enterolobii* populations (R1, R4, R5 and R6) grouped in a first cluster, while *M. javanica* populations (R24, R27, R28, R30 and R31) were placed in a second. This distinction was very clear and the differences between these two species (first dimension in the PCA) explained 89.7% of the variation in the dataset (Fig. 1A). Although the two populations of *M. incognita* (R25 and R34) exhibited different genetic relation on this first dimension, this species was clearly separated from *M. enterolobii* and *M. javanica* in the second dimension of the PCA explaining 4.1% of the total variation (Fig. 1B).

**Figure 2:**
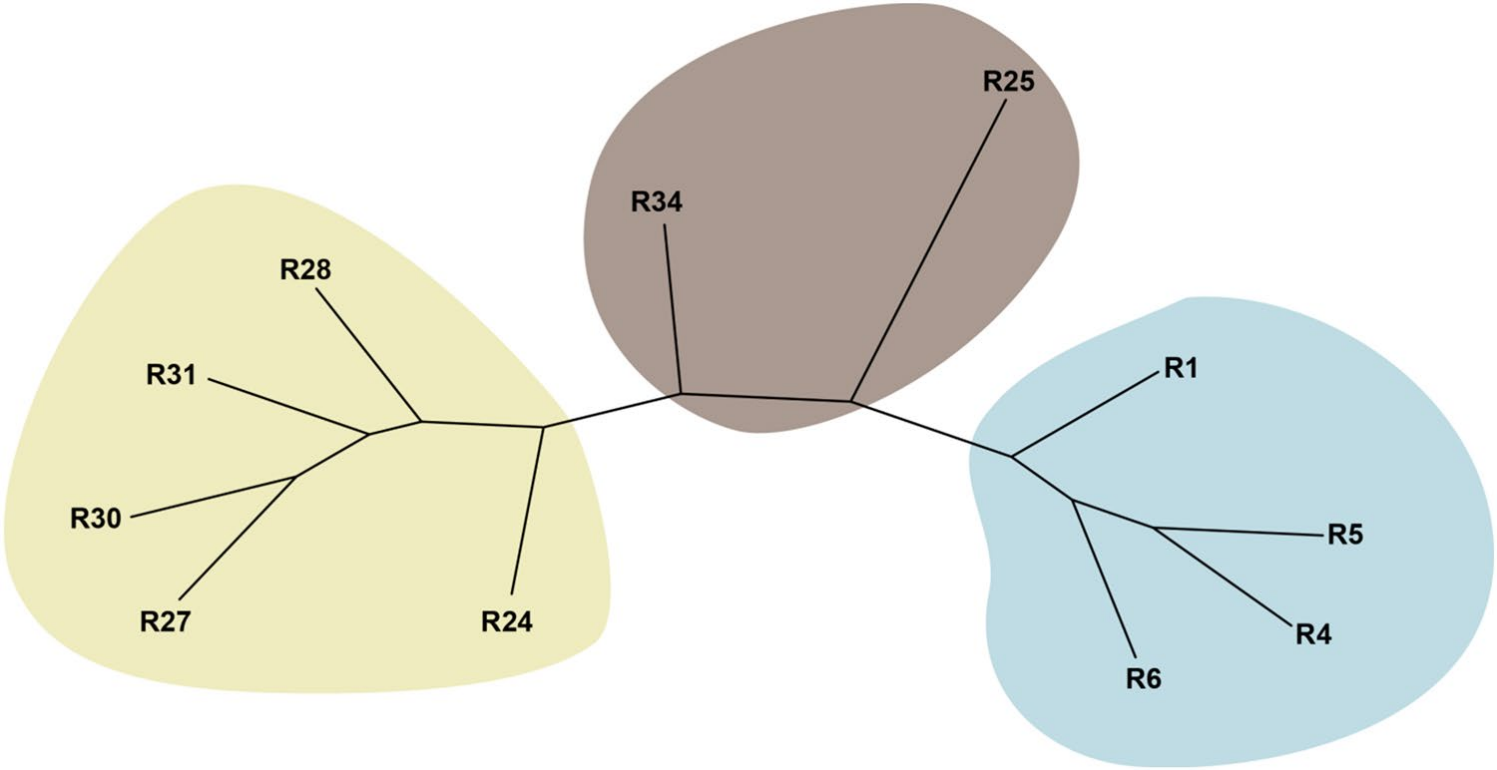
Neighbor-joining phylogenetic tree of 11 South African *Meloidogyne* populations, representing two *M. incognita* (brown), four *M. enterolobii* (blue) and five *M. javanica* (beige) populations, based on genome-wide allele frequencies at 277 loci.

The phylogenetic tree, using the dataset of 277 SNPs, also revealed two main clusters separating *M. enterolobii* populations from those of *M. javanica*, with *M. incognita* populations being intermediate (Fig. 2).

A direct comparison of allele frequencies between species highlighted several SNPs clearly differentiating the three species. Among these, three had different zygosity status between the species in all the populations tested and 31 were homozygous for an allele only in *M. enterolobii* (Table 2). The sequence surrounding each SNP has been retrieved from the *M. incognita* reference genome. Out of these, 19 were located in predicted genes. When screening the variants obtained by aligning the raw reads to the *M. incognita* genome, 14 were located in genes coding for 10 different proteins and had allele frequencies specific to *M. enterolobii* (Table 3).

**Table 2:** Homozygous loci in *Meloidogyne enterolobii*, obtained by genotyping by sequencing, that putatively discriminate the species from two other *Meloidogyne* species from South Africa.

Locus	Alleles	Allele frequency^a^			Localization or gene function
		*M. enterolobii*	*M. incognita*	*M. javanica*	
*Discriminating all 3 species*					
TP40058	G/A	1	0.37	0	Muscle M-line assembly protein unc-89
TP37437	T/C	1	0.56	0	n.a.^b^
TP30509	T/A	0	0.69	1	n.a.
*Homozygous in Me* ^c^ *and different in other species*					
TP11533	T/A	1	0.50	0.06	uncharacterized protein (CBN07616) *Caenorhabditis brenneri*
TP1752	T/C	0	0.60	1	Histone deacetylase
TP17550	A/T	0	0.08	0.08	n.a.
TP22081	T/C	0	0.55	1	Zinc finger, C2H2
TP27938	T/C	0	0.58	1	intergenic
TP27939	T/C	0	0.94	1	intergenic
TP27941	T/C	0	0.55	0.99	n.a.
TP28969	G/A	1	0.43	0	vacuolar protein sorting-associated protein VTA1
TP30499	T/A	0	0.56	1	n.a.
TP34022	T/G	1	0.38	0.20	n.a.
TP34025	G/T	1	0.33	0.25	cytoplasmic polyadenylation element-binding protein
TP35049	G/A	0	0.53	0.63	n.a.
TP35052	G/A	0	0.49	0.46	spectrin beta, non-erythrocytic 4^d^
TP35086	G/A	0	0.47	0.45	n.a.
TP35088	G/A	0	0.47	0.53	Plectin repeat^e^
TP36654	G/A	0	0.60	1	Valine—tRNA ligase
TP36738	G/A	0	0.54	0.99	Valine—tRNA ligase
TP37436	T/C	1	0.85	0.03	Unknown
TP4522	T/C	1	0.48	0.02	Intergenic
TP555	G/A	0	0.14	0.81	n.a.
TP558	G/A	0	0.23	0.82	n.a.
TP7297	G/C	1	0.67	0.26	intron
TP9881	G/A	0	0.65	1	n.a.

^a^Allele frequencies represent the mean of four populations for *M. enterolobii*, two for *M. incognita* and five for *M. javanica*. ^b^n.a. indicated that this sequence was not retrieved from the reference genome. ^c^*Meloidogyne enterolobii*. ^d^Two similar SNPs (TP35055 and TP35079) were found in this protein and probably represent gene duplication or alignment artefacts. ^e^Six similar SNPs (TP35091, TP35096, TP35115, TP35118, TP35125 and TP35129) were found in this protein and probably represent gene duplication or alignment artefacts.

**Table 3:** Homozygous loci in *Meloidogyne enterolobii*, obtained by alignment of reads on the *M. incognita* reference genome that discriminate the species from the other selected *Meloidogyne* species from South Africa, and that are located in annotated genes.

Locus	Alleles	Allele frequency^a^			Description	E value
		*M. enterolobii*	*M. javanica*	*M. incognita*		
MiV1ctg11_241891	G/A	0	1	1	fibronectin type III domain	5.71e-23
MiV1ctg17_134782	T/G	0	1	0.52	Ubiquitin carboxyl-terminal hydrolase 4	2.95e-07
MiV1ctg2_298920	T/C	0	0.29	0.67	Major facilitator superfamily MFS-1 domain containing	4.31e-16
MiV1ctg27_10072	A/T	0	0.97	0.52	Serine threonine- phosphatase 2B catalytic subunit	2.25e-05
MiV1ctg27_9897	T/C	0	0.99	0.52	Serine threonine- phosphatase 2B catalytic subunit	2.25e-05
MiV1ctg39_178906	A/G	0	0.58	0.59	Cytoskeleton-associated 5	1.43e-11
MiV1ctg39_178981	T/A	1	0.04	0.35	Cytoskeleton-associated 5	1.43e-11
MiV1ctg59_26757	A/G	0	1	0.53	Glycogenin-1	1.95e-25
MiV1ctg59_26792	A/G	0	1	0.53	Glycogenin-1	2.45e-18
MiV1ctg61_132221	ATCAA/ACAG	0	1	0.67	sodium- and chloride-dependent glycine transporter 1	2.46e-16
MiV1ctg7_255261	C/T	1	0.71	0.22	choline Carnitine O-acyltransferase	1.07e-12
MiV1ctg7_255276	G/T	0	1	0.57	choline Carnitine O-acyltransferase	1.23e-12
MiV1ctg7_56934	T/C	0	1	0.89	Valyl-tRNA synthetase	3.06e-49
MiV1ctg75_84149	A/C	0	1	0.83	adenylate kinase isoenzyme 5	1.13e-16

^a^Allele frequencies represent the mean of four populations for *M. enterolobii*, two for *M. incognita* and five for *M. javanica*.

## Discussion

This study represents a baseline investigation of the genetic diversity of South African *Meloidogyne* populations. Root-knot nematodes are reported to infect various crop hosts and to cause great damage and economic losses in South Africa. Since *M. enterolobii* is known to be highly virulent, and because resistant cultivars are not equally effective against the different *Meloidogyne* species, accurate and reliable species identification is crucial to use appropriate management strategies.

The GBS method used in this study proved to be useful in identifying diagnostic SNPs for the discrimination of three of the highly damaging thermophilic *Meloidogyne* species occurring in South Africa. Accurate distinction of *M. enterolobii*, *M. javanica* and *M. incognita* was still challenging using various molecular techniques, except for SCAR-PCR^20^. Although *M. enterolobii* can be separated from other *Meloidogyne* species by the use of various universal markers e.g. 28S, COI, 16S and IGS^24,37,38^, distinguishing between *M. javanica* and *M. incognita* has been unsuccessful in various studies due to the high genetic similarity among the latter species and *M. arenaria*^37–41^. In this study, however, several putatively discriminative SNPs to *M. javanica, M. incognita* and *M. enterolobii* were identified and will enable the accurate distinction between these species through the development of allele-specific PCR. The good number of SNPs obtained with UNEAK on the *M. enterolobii* populations alone revealed the high potential of this method and indicated that the technique will be very useful to compare more populations of this species and to include co-variables like virulence or origin for a more in-depth characterization. Ultimately, this kind of comparison should be done using whole genome sequencing. However, when dealing with numerous samples, the cost associated with WGS is still prohibitive. Thus, GBS represents an interesting alternative. This molecular approach was initially described for plants by Elshire *et al*.^31^, and modified specifically for nematodes by Mimee *et al*.^27^. It was used for the first time during this study to investigate the genetic diversity in *Meloidogyne* species. The technique also takes advantage of Pool-Seq (sequencing of composite samples) which removed the fastidious step of isolating and extracting DNA from single juveniles. The bioinformatics pipeline allowed the rapid *de novo* identification of SNPs, without the need of a reference genome. When aligning the sequencing reads to the closest reference species, *M. incognita*, we found less good quality SNPs (no missing data and good coverage) than when using the GBS pipeline. This indicates that significant differences exist between the two species. This was confirmed by the eight-fold increase in the number of SNPs when the pipeline was run with *M. enterolobii* populations alone. As UNEAK will only keep the SNPs that are present in all populations (at minimum call rate = 1.0), this approach indicates that the three species are probably more different than anticipated. On the other hand, some genetic variants were found upon alignment with the *M. incognita* genome and not by the UNEAK pipeline. This is explained by the high stringency of the pipeline that only tolerates one mismatch by read and rejects sequences with multiple SNPs or other kind of variants. Therefore, combining the two approaches will maximize the discovery rate of SNPs in the genome of *Meloidogyne* species. Also, it was hypothesized that several mitotic parthenogens *Meloidogyne* species had acquired pairs of divergent gene copies during past hybridisation event^35^. This will result in an excess of heterozygosity that could complicate classical phylogenetic analyses. One of the advantage of GBS is that it is not affected by ploidy as the UNEAK pipeline compare all the sequences. Thus, if the same sequence is present in many versions in an organism due to ploidy or gene duplication, the pipeline will compute all versions together and output the allele frequency for that sequence and not for each physical locus in the genome. Furthermore, using Pool-Seq, we theoretically captured all the allelic diversity of each population.

PCA analyses confirmed the genetic separation between *M. enterolobii, M. javanica* and *M. incognita*. This result was expected since the distinction of these species has been reported using different markers^24,37^. The analysis also revealed a close genetic proximity of the four *M. enterolobii* populations when the 277 SNPs dataset (present in all four populations) was used. This is in agreement with results obtained by Tigano *et al*.^22^ that described *M. enterolobii* as a geographic homogenous species with low diversity between populations. Similarly, Onkendi and Moleleki^38^ showed 100% homology between sequences of IGS and COII from South African *M. enterolobii* populations. However, when the GBS approach was used for *M. enterolobii* only, more variation was observed and suggests that this species could be more diverse than initially thought. As genetic diversity is an important driver of adaptation to new environmental conditions or host plants, it will be interesting to explore this diversity by studying more *M. enterolobii* populations.

When comparing allele frequencies of 277 SNPs distributed all across the genome, the two populations of *M. incognita* investigated in this study showed substantial genetic difference, one being related to *M. enterolobii* and the other to *M. javanica*. A possible explanation for this phenomenon may be that in this analysis only loci sequenced in all *Meloidogyne* populations were conserved, meaning that the sequences that were unique to one or two species only (not present or not sequenced in the other) were discarded even if they contain SNPs. Thus, this place the emphasis on the differences that exist in terms of SNPs contained in the genomes of these three *Meloidogyne* species.

This study also highlighted allelic differences between the species that exists in specific genes that could modify protein sequence and function. Ultimately, in depth characterization of the *M. enterolobii* genome will give us a better understanding of why the species is more aggressive and overcome resistance genes that are effective against *M. incognita* and *M. javanica*. For now, 19 SNPs identified by UNEAK and 14 by the alignment on the *M. incognita* reference genome were located in exons of predicted genes. These variants were specific to *M. enterolobii* and could affect nematode-host interactions. Some of these genes have already been reported to be involved in parasitism. For example, two SNPs were located in genes coding for a serine/threonine phosphatase. A protein with a similar sequence is a known effector in the Hessian fly, *Mayetiola destructor*. This plant-galling arthropod uses effectors to modify host cells in a way that is superficially similar to root-knot nematodes. The type-2 serine/threonine protein phosphatase (PP2C) domain was shown to be associated with the ability of the fly to survive and parasite wheat seedlings in susceptible plants. This protein is also recognized as an avirulence factor in cultivars carrying the *H24* resistant genes. Interestingly, a single loss-of-function mutation in that gene is sufficient to overcome resistance in virulent population^42^.

Results from this study also highlighted differences between *M. enterolobii* and the other species in a sequence coding for a zinc finger, C2H2 domain. These small protein motifs are known to interact with different molecules and are involved in multiple functions from gene transcription and mRNA trafficking to protein folding and apoptosis^43^. Wang *et al*.^44^, reported that the C2H2 zinc finger *PsCZF1* was involved in pathogenesis in *Phytophthora sojae* by demonstrating that *PsCZF1*-deficient mutants lost virulence on different soybean cultivars. Another study on *Alternaria brassicicola* showed that a knockdown mutation in the zinc finger transcription factor *AbVf19* resulted in a 90% decrease in virulence^45^. Interestingly, Gross and Williamson^46^ identified a novel transposable element in root-knot nematodes that contained a C2H2 zinc-finger motif and that could be involved in the ability of some populations to bypass resistance in tomato mediated by the *Mi-1* gene.

Finally, the plant cytoskeleton is hypothesized to play a crucial role in host defense response and a target for virulence^47^. Root-knot nematodes are actively remodeling the cytoskeleton of infected plants during feeding cell and gall formation^48^. This reprogramming is induced by secreted effectors, several being homologous to plant proteins^49^. In this study, we have identified mutations in several *M. enterolobii* genes that code for proteins required for microtubule and cytoskeleton formation: Cytoskeleton-associated 5, spectrin and plectin repeat.

In this paper, the GBS technique proved to be a powerful and accurate technique to obtain detailed information about the diversity that exists among root-knot nematode species. The bioinformatic pipeline allowed the identification of high-quality diagnostic SNPs from the South African *Meloidogyne* species. The phylogenetic comparison of these variants generated valuable and novel knowledge about the genetic diversity of *Meloidogyne* species. Candidate genes associated with virulence were also highlighted and should be further explored to evaluate whether they are involved in *M. enterolobii* pathogenicity.

## Methods

### Sampling and DNA extraction

Eleven *Meloidogyne* populations, representing *M. enterolobii, M. incognita* and *M. javanica* (Table 4), were obtained from root and rhizosphere soil of different host plants in the Mpumalanga and Limpopo provinces of South Africa during 2015 and 2016. Single egg masses of each population were inoculated on roots of two-leaf stage tomato seedlings of a root-knot nematode susceptible cultivar (Floradade)^50^ to ensure species purity. Subsequent mass rearing of pure populations was done in the glasshouse on tomato seedlings (cultivar Floradade) planted in individual pots that were filled with sandy-loam soil (5.3% clay, 93.6% sand, 1.1% silt, 0.47% organic matter and pH (H_2_O) of 7.47) previously fumigated with Telone II (as. 1,3-dichloroproene; dosage of 150 l/ha). An ambient temperature range of 21 (min) – 26 °C (max) and 14L:10D photoperiod were maintained in the glasshouse. Sixty days after inoculation, approximately 100 females of each population were randomly dissected from infected tomato roots of individual plants and DNA extracted by using DNeasy Blood & Tissue kit (Qiagen, Germany) according to the protocol supplied. The DNA of each *Meloidogyne* population was quantified using the Invitrogen Quant-iT Qubit dsDNA HS Assay Kit (Invitrogen, USA), and 30 ng of each sample was used for sequencing library construction.

**Table 4:** Root-knot nematode species used in this study as well as the origin of the species and host plants which it infected.

Sample ID	Nematode Species	Locality of origin	Host Plant
R1	*M. enterolobii*	Mbombela 1 (Mpumalanga)	Guava: *Psidium guajava*
R4	*M. enterolobii*	Hoedspruit (Limpopo)	Guava: *Psidium guajava*
R5	*M. enterolobii*	Erik Boerdery (Mpumalanga)	Guava: *Psidium guajava*
R6	*M. enterolobii*	Erik Boerdery (Mpumalanga)	Guava: *Psidium guajava*
R25	*M. incognita*	Mooketsi 1(Limpopo)	Tomato: *Solanum lycopersicum*
R34	*M. incognita*	Pont Drift (Limpopo)	Tomato: *Solanum lycopersicum*
R24	*M. javanica*	Mbombela 2 (Mpumalanga)	Tomato: *Solanum lycopersicum*
R27	*M. javanica*	Mooketsi 2 (Limpopo)	Tomato: *Solanum lycopersicum*
R28	*M. javanica*	Mooketsi 3 (Limpopo)	Tomato: *Solanum lycopersicum*
R30	*M. javanica*	Polokwane (Limpopo)	Tomato: *Solanum lycopersicum*
R31	*M. javanica*	Mbombela 3 (Mpumalanga)	Spinach: *Spinacia oleracea*

### Species identification

All populations of *Meloidogyne* species used in this study were identified using morphological and molecular approaches. Morphological identification was done based on female oesophagus and perineal patterns. The molecular confirmation was done by sequencing the 28S rDNA (D2-D3)^51^, COI^52^ and NADH5^37^ mtDNA, regions and comparing to NCBI database, and by the amplification of a species specific SCAR marker^20,53^.

### Library preparation and sequencing

Sample preparation and sequencing were done by the Genomics Analysis Core Facility at the Institute for Integrative and Systems Biology (IBIS; Université Laval, Quebec, Canada) according to the GBS method developed by Elshire *et al*.^31^, using *Pst*I/*Msp*I restriction enzymes. The library was sequenced on one Ion Proton chip (ThermoFisher Scientific) at IBIS.

### SNP calling and processing of the raw sequences

The Universal Network Enabled Analysis Kit (UNEAK) pipeline^54^ was used to analyse Pool-Seq data and create the SNP list for the 11 *Meloidogyne* populations. To eliminate sequencing errors, a tolerate rate of 0.03 was used for UNEAK running. Filtering of SNPs was done by setting the minimum call rate (number of populations in which a locus must have been scored) at 0.8 (i.e. <20% missing data) or 1.0 (no missing data). The minimum minor allele frequency (MAF) threshold was set to 0.01. The effect of locus coverage on the final SNP number was also compared by setting the minimum number of reads at a given locus in each population required to accept a SNP (minCov) to 5, 20, or 50. The UNEAK pipeline was also run on the *M. enterolobii* populations alone with the minimum call rate set at 0.7 or 1.0 and the minimum coverage at 5, 20 and 50.

Raw sequences were also aligned on the *M. incognita* genome^3^ by using burrows-wheeler aligner (BWA)^55^ after trimming with Trimmomatic (TRAILING = 20, MINLEN = 20) (http://www.usadellab.org/cms/?page=trimmomatic) and demultiplexing with Sabre (https://github.com/najoshi/sabre). Then, variants and annotations were called with freebayes^56^ and SnpEff^57^ respectively using the gene-finding format (GFF) of *M. incognita*.

### Population genetics

A neighbor-joining phylogenetic tree was elaborated by using Gendist and Neighbor modules in PHYLIP v3.695 with the genome-wide allele frequencies obtained from UNEAK. PCA was done in R by using the same sets of allele frequencies with the *prcomp*() function from the *stats* package.

The SNPs of interest from UNEAK were retrieved from the *M. incognita* genome by means of BLASTN with the default parameters, except for a smaller word size of 4, with the Blast2GO application^58^.

## Electronic supplementary material

Supplementary Table 1

## Data Availability

The data are submitted to NCBI SRA Portal with the following information. BioProject (PRJNA485255) and accession number (SAMN09786892, SAMN09786893, SAMN09786893, SAMN09786895, SAMN09786896, SAMN09786897, SAMN09786898, SAMN09786899, SAMN09786900, SAMN09786901 and SAMN09786902).
